# Effects of seedling age at transplanting on root-shoot traits and yield in direct-seeded maize

**DOI:** 10.1371/journal.pone.0352438

**Published:** 2026-07-15

**Authors:** Xin Gao, Jie Peng, Hongwei Liang, Yujie Liu, Junyang Qiao, Xiaolong Liu

**Affiliations:** 1 College of Agriculture, Inner Mongolia Minzu University, Tongliao, China; 2 Inner Mongolia Forage Crop Engineering Technology Research Center, Tongliao, China; 3 Institute of Maize Research, Inner Mongolia Academy of Agricultural & Animal Husbandry Sciences, Huhehaote, China; Ardakan University, IRAN, ISLAMIC REPUBLIC OF

## Abstract

To address weak growth and low yield of spring maize under transplanting and seedling replacement, this study evaluated the effects of transplanting at different seedling ages on dry matter production, with the aim of identifying the optimal transplanting stage. Field experiments were conducted at two sites using a randomized block design. Three transplanting stages—2-leaf 1-heart (2L1H), 3-leaf 1-heart (3L1H), and 4-leaf 1-heart (4L1H)—were compared with direct seeding (CK). Root shoot traits, dry matter accumulation, and ear characteristics were assessed. Compared with direct seeding, transplanting shortened the jointing-to-silking period but extended both the transplanting-to-jointing and silking-to-maturity phases. Transplanting significantly reduced root traits, including 0–20 cm root dry mass(RDM), root length(RL), root surface area(RS), and number of forks (NF), as well as leaf traits such as leaf area(LA), specific leaf weight (SLW), and SPAD values. Transplanted plants exhibited physiological stress, reflected by decreased leaf instantaneous spectral use efficiency (SUE) and increased water use efficiency (WUE) and instantaneous carboxylation rate (Ce). These changes limited dry matter accumulation and its translocation to grains, resulting in reduced yield. Among the treatments, transplanting at the 3-leaf 1-heart stage (3L1H) showed the smallest deviation from direct seeding in root traits and dry matter accumulation, thereby minimizing yield loss.

## 1. Introduction

As one of the world’s three major staple crops, maize has long exceeded its traditional role as a food source [1,2]. As an important raw material for livestock feed, pharmaceuticals, industrial products, and biofuel production, maize is closely integrated with global economic and ecological systems and plays a vital role in food security and economic development [3]. As the world’s second-largest maize consumer, China prioritizes yield improvement as a central objective of maize production. Increasing planting density is an effective agronomic strategy to achieve higher yields [4–6]. However, factors such as seed quality, mechanical sowing performance, and soil moisture conditions often result in missing seedlings and uneven plant stands, limiting the realization of optimal planting density [7,8]. To mitigate yield loss, farmers commonly adopt on-site transplanting of direct-seeded seedlings to fill gaps in the field. In this practice, excess seedlings are removed from densely sown areas after emergence and transplanted into missing-plant positions. However, this method often results in weak plants, underdeveloped ears, low seed set, and even barren stalks, ultimately constraining yield formation. Therefore, investigating the effects of transplanting direct-seeded seedlings on maize growth, development, and yield formation is of practical significance for field production.

At present, research on transplanting mainly focuses on seedling cultivation and transplanting, in which seedlings are raised at high density in specialized nurseries (e.g., trays or seedbeds) and then transplanted to the field at a defined stage. Compared with on-site transplanting, this method requires higher initial investment and is more suitable for large-scale production [9]. Seedling cultivation and transplanting have a long history, with the earliest record transplanting was in the *Book of Fan Sheng* in the Western Han Dynasty of China 2,000 years ago. Today, this technique is widely used in various crops to improve yield and enhance resource use efficiency [10]. In maize production, transplanting can regulate growth and optimize population structure, and is often used to advance planting schedules or extend the growth period, thereby improving the utilization of light and thermal resources and contributing to stable, high yields. However, improper transplanting practices may disrupt plant growth and negatively affect development [11–13]. Among the factors influencing transplanting success, seedling age plays a critical role. Appropriate transplanting age can improve crop growth, yield, and quality, whereas unsuitable timing can significantly reduce yield [14,15]. As seedling age increases, the growth period is prolonged, while leaf area index, photosynthetic potential, and photosynthetic rate tend to decline. Yield components, including ear length, ear number, and grain number, also decrease, ultimately resulting in reduced grain yield [16,17]. Compared with direct seeding, transplanting may increase dry matter accumulation, harvest index, and water and nitrogen use efficiency. However, it can also reduce leaf area, intercepted photosynthetically active radiation, radiation use efficiency, and grain quality parameters (e.g., crude protein, starch, ash, crude fiber, and oil content), with significant varietal differences observed [18,19].

Although many studies have examined seedling transplanting, relatively few have focused on the on-site transplanting of direct-seeded maize seedlings, particularly in relation to root crown characteristics and yield formation. Therefore, this study conducted field experiments based on direct-seeded maize transplanting at different seedling ages. By analyzing root morphological characteristics, leaf traits, photosynthetic performance, dry matter accumulation, and yield responses, this study aimed to clarify the effects of transplanting timing on root crown characteristics and yield formation in spring maize, and to provide a theoretical basis for improving on-site seedling replacement practices.

## 2. Materials and methods

### 2.1. Experimental design

The study was conducted at two experimental sites in 2024. Experiment Site 1 was located in Wenduhua Village, Fengtian Town, Keerqin District, Tongliao City, Inner Mongolia (hereafter referred to as Wenduhua), situated at 122°41′E, 43°91′N. The site has an average annual temperature of 6.6 °C, average annual precipitation of 373.6 mm, and annual sunshine duration of 2500 ~ 2800 h. The soil type is sandy soil, with organic matter content of 8.55 g/kg, available nitrogen of 30.06 mg/kg, available phosphorus of 11.05 mg/kg, and available potassium of 75.23 mg/kg in the 0 ~ 20 cm soil layer. Experiment Site 2 was located in Dongsheng Village, Liaohe Town, Tongliao Economic and Technological Development Zone, Inner Mongolia (hereafter referred to as Dongsheng), situated at 122°18′E, 43°73′N. The site has an average annual temperature of 6.6 °C, average annual precipitation of 458.8 mm, and annual sunshine duration of 2500 ~ 2800 h. The soil type is meadow soil, with organic matter content of 18.27 g/kg, available nitrogen of 59.12 mg/kg, available phosphorus of 7.17 mg/kg, and available potassium of 126.48 mg/kg in the 0 ~ 20 cm soil layer.

**Fig 1:**
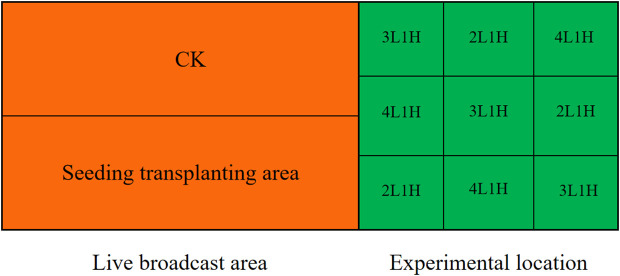
Field layout diagram.

The field experiment at both sites adopted a randomized block design with three transplanting stages: 2-leaf 1-heart (2L1H), 3-leaf 1-heart (3L1H), and 4-leaf 1-heart (4L1H). Each treatment was replicated three times, resulting in a total of nine plots, each with an area of 18 m². Direct seeding was used as the control (**Fig 1**). Sowing was carried out at the Wenduhua site on 3 May using the spring maize cultivar Dika159 and at the Dongsheng site on 6 May using the spring maize cultivar Zhongnongda 787. Both cultivars are locally recommended and have similar growth durations. Both experimental sites adopted a wide–narrow row planting pattern (80 cm + 40 cm), with a planting density of 7.5 × 10⁴ plants ha ⁻ ¹. A single basal application of slow-release fertilizer (N:P:K = 28:12:12) was applied at 750 kg ha ⁻ ¹ for all treatments. Drip irrigation was used with a total irrigation quota of 3600 m³ ha ⁻ ¹. Weeds and pests were effectively controlled throughout the growing season.

### 2.2. Field sampling and trait measurements

#### 2.2.1. Plant leaf area and dry matter accumulation.

During the silking stage, three representative maize plants with uniform growth were selected from each plot. The plants were cut at the stem base, and stems were separated from leaves. Leaf length (L) and width (W) were measured. Leaf area (LA) was calculated using the length–width coefficient method as follows:


$$\begin{array}{c} \text{LA}=0.75\times \sum_{i=1}^{u}{\text{L}}_{i}\times {\text{W}}_{i} \end{array}$$

The sampled leaves and non-leaf plant organs were first oven-dried at 105 °C for 30 min and then dried to constant weight at 80 °C in a forced-air oven. Leaf dry mass (LDM) and non-leaf organ dry mass (NLDM) were recorded, and total plant dry matter (DM) was calculated as the sum of all components.Specific leaf weight (SLW) was calculated as leaf dry mass (LDM) divided by leaf area (LA): SLW = LDM / LA. At physiological maturity, dry matter sampling was conducted using the same procedure as at the silking stage. Grain dry mass and non-grain organ dry mass were then measured.

#### 2.2.2. Relative chlorophyll content (SPAD value).

During the silking stage, three representative maize plants with uniform growth were selected from each plot. The SPAD values of the upper leaves (top three leaves), middle leaves (at the ear position), and lower leaves (near the ground) were measured using a portable SPAD-502PLUS chlorophyll meter. Five measurement points were evenly selected on each leaf. The mean value of the three leaves was calculated as the SPAD value for each leaf position. The overall plant SPAD value was calculated as the average of the values from the upper, middle, and lower leaf positions.

#### 2.2.3. Photosynthetic indices.

During the silking stage, three representative maize plants with uniform growth were selected from each plot. Between 9:00 and 12:00 a.m., a portable photosynthesis system (LI-6400, LI-COR Biosciences) was used to measure net photosynthetic rate (Pn), transpiration rate (Tr), intercellular CO₂ concentration (Ci), and photosynthetically active radiation (PAR) in the upper leaves, middle leaves, and lower leaves.Photosynthetic efficiency parameters were calculated as follows: Instantaneous spectral use efficiency (SUE) = Pn / PAR × 100%; Instantaneous water use efficiency (WUE) = Pn / Tr; Instantaneous carboxylation efficiency (Ce) = Pn / Ci. At the plant level: Plant spectral use efficiency (SUEₚₗₐₙₜ) = SUE × LA; Plant water use efficiency (WUEₚₗₐₙₜ) = WUE × LA; Plant carboxylation efficiency (Ceₚₗₐₙₜ) = Ce × LA.

#### 2.2.4. Root traits.

During the silking stage, three representative plants were selected from each plot. Roots from the 0 ~ 20 cm, 20 ~ 40 cm, and 40 ~ 60 cm soil layers were carefully excavated. Root length(RL), average diameter(AD), root surface area(RS), and number of forks(NF) were measured using a RHIZO 2009 root analysis system. After scanning, the roots were oven-dried at 80 °C to constant weight and then weighed and recorded(RDW).

#### 2.2.5. Root/shoot ratio.

At different transplanting seedling stages, 10 representative plants with uniform growth were selected for whole-plant sampling. After washing, each plant was separated into aboveground and belowground components. Samples were oven-dried to constant weight and weighed separately. The root/shoot ratio (R/S) at the silking stage was calculated using the following formula: R/S = RDW / DM.

#### 2.2.6. Ear traits.

At physiological maturity, 10 representative ears with uniform growth were selected from each plot. Ear length(EL), ear diameter(ED), ear row number(ERN), number of kernels per row(KNR), 1000-kernel weight, and single-ear kernel weight (KW) were measured.

#### 2.2.7. Data processing and statistical analysis.

Data processing and analysis were conducted using Microsoft Excel 2016. Analysis of variance (ANOVA) was performed using IBM SPSS Statistics 19.0 software. Significant differences among treatments were determined using the least significant difference (LSD) test at a significance level of *P* < 0.05. Correlation analysis and graphical plotting were carried out using Origin 2019 software.

## 3. Results

### 3.1. Analysis of variance

As shown in **Table 1**, analysis of variance (ANOVA) was conducted on maize phenotypic traits under different transplanting stages. Experimental location and transplanting treatment both had significant effects on root, shoot, and yield-related traits, with most traits showing significant differences. However, the interaction between location and treatment had a relatively limited effect on these traits. Only RDW (20 ~ 40 cm), RDW (40 ~ 60 cm), RDW(20 ~ 40 cm)、RDW(40 ~ 60 cm)、RL、RS、WUEₚₗₐₙₜ、Ceₚₗₐₙₜ、KW showed significant interaction effects.

**Table 1:** Analysis of variance for root and shoot traits and yield indices in maize(F-value).

Sources	RDW(0~20cm)	RDW(20~40cm)	RDW(40~60cm)	RL	RS	AD	NF
Site(S)	16.35*	86.29*	4.14	94.07*	82.36*	0.66	326.40*
Treatment(T)	102.91*	71.24*	21.16*	67.29*	63.42*	1.37	57.42*
S×T	0.19	63.52*	21.85*	5.44*	5.05*	0.10	1.55
Source	LA	SLW	**SPAD values**				DM
			**(Upper)**	**(Middle)**	**(Lower)**	**(Whole)**	
Site(S)	597.35*	63.23*	0.45*	97.24	134.91*	72.35*	7.62*
Treatment(T)	30.77*	13.98*	5.93*	2.32	4.09*	5.74*	36.75*
S×T	1.39	2.23	0.22	0.16	1.99	0.22	0.9
Source	SUE	WUE	Ce	SUE_plant_	WUE_plant_	Ce_plant_	LDM
Site(S)	71.26	95.98*	821.59*	551.25*	629.23*	78.55*	85.53*
Treatment(T)	71.77*	100.22*	300.82*	130.18*	49.19*	191.59*	37.23*
S×T	0.81	11.61*	42.48*	1.52	22.00*	78.76*	1.29
Sources	NLDM	EL	ED	ERN	KNR	1000-KW	KW
Site(S)	1.63*	2.78	0.12	0.60	6.20*	0.19	41.66*
Treatment(T)	22.05*	57.60*	21.93*	0.42	11.74*	34.23*	308.76*
S×T	0.84	0.23	1.04	1.09	0.68	0.40	10.19*

RDW, root dry weight; RL, root length; RS, root surface area; AD, average diameter; NF, number of forks; LA, leaf area; SLW, specific leaf weight; SPAD values, relative chlorophyll content; DM, dry matter; SUE, instantaneous spectral use efficiency; WUE, instantaneous water use efficiency; Ce, instantaneous carboxylation efficiency; SUE_plant_, plant spectral use efficiency; WUE_plant,_ plant water use efficiency; Ce_plant_, plant carboxylation efficiency; LDM, leaf dry mass; NLDM, non-leaf organ dry mass; EL, ear length; ED, ear diameter; ERN, ear row number; KNR, number of kernels per row; 1000-KW, 1000-kernel weight; KW, single-ear kernel weight. * indicate significance at p < 0.05.

### 3.2. Effect of seedling transplanting age on growth period

Seedling transplanting delayed maize phenological development and extended the overall growth period, with older seedlings generally showing longer growth durations (**Table 2**). Compared with the control (CK), transplanting at the 2-leaf 1-heart stage (2L1H) delayed the jointing stage by 4 days, tasseling stage by 2 ~ 3 days, silking stage by 3 days, milk stage by 2 ~ 3 days, and physiological maturity by 6 ~ 7 days. The total growth period was extended by 6 ~ 7 days. The duration from jointing to silking was shortened by 2 days, while the period from silking to maturity was extended by 4 days. At the 3-leaf 1-heart stage (3L1H), jointing was delayed by 7 ~ 8 days, tasseling by 4 ~ 5 days, silking by 5 days, milk stage by 4 ~ 6 days, and maturity by 8 ~ 9 days. The total growth period was extended by 8 ~ 9 days. The jointing-to-silking period was shortened by 2 ~ 3 days, whereas the silking-to-maturity period was extended by 4 ~ 5 days. At the 4-leaf 1-heart stage (4L1H), jointing was delayed by 11 ~ 12 days, tasseling by 7 ~ 8 days, silking by 9 ~ 10 days, milk stage by 6 ~ 8 days, and maturity by 11 days. The total growth period was extended by 11 days. The jointing-to-silking period was shortened by 2 days, while the silking-to-maturity period was extended by 1 ~ 2 days. Overall, these results indicate that within a certain seedling age range, older transplanting seedlings shorten the vegetative phase (jointing to silking) while extending the reproductive phase. However, when transplanting is delayed beyond a certain threshold (≥4L1H), the extension of the reproductive growth period is reduced.

**Table 2:** Investigation and Record of the Growth Stage.

Experimental location	Treatment	Sowing time	Transplanting period	Jointing stage (V6)	Bell-mouthing stage (V12)	Silking (R1)	Milking (R3)	Maturity period (R6)
Wenduhua	2L1H	5/3	5/19	6/29	7/10	7/25	8/17	10/1
	3L1H	5/3	5/21	7/2	7/12	7/28	8/19	10/3
	4L1H	5/3	5/24	7/6	7/15	8/1	8/21	10/6
	CK	5/3	－	6/25	7/8	7/23	8/15	9/25
Dongsheng	2L1H	5/6	5/21	7/2	7/13	7/27	8/21	10/4
	3L1H	5/6	5/24	7/6	7/15	7/30	8/24	10/6
	4L1H	5/6	5/27	7/10	7/18	8/4	8/27	10/8
	CK	5/6	－	6/28	7/10	7/25	8/18	9/27

2L1H,2-leaf 1-heart; 3L1H, 3-leaf 1-heart; 4L1H, 4-leaf 1-heart; indicating three transplanting stages; CK, direct seeding. The same as below.

### 3.3. Effect of field transplanting timing on root traits

**Fig 2:**
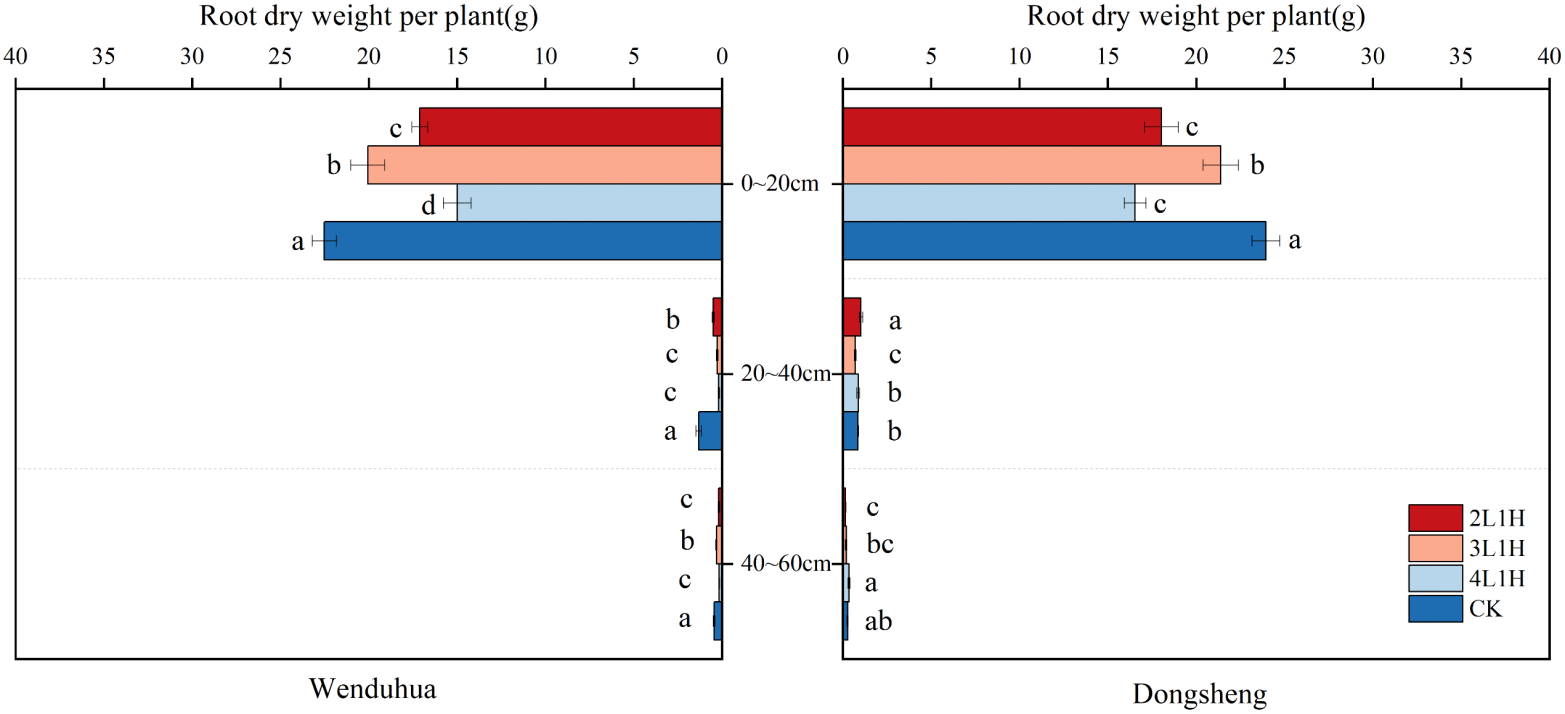
Effects of seedling transplanting age on root dry weight in maize plants. The middle vertical coordinate represents the depth of the soil layer. Different letters above bars indicate significant differences between treatments (p < 0.05).

As shown in **Fig 2**, more than 90% of root dry weight was distributed in the 0 ~ 20 cm soil layer. Transplanting at the seedling stage significantly reduced root dry weight in this layer. At the Wenduhua site, root dry weight in the 0 ~ 60 cm soil profile was significantly lower in all transplanting treatments compared with the control (CK). In the 0 ~ 20 cm layer, root dry weight decreased by 23.98%, 10.90%, and 33.43% under the 2L1H, 3L1H, and 4L1H treatments, respectively. In the 20 ~ 40 cm layer, it decreased by 61.73%, 79.59%, and 86.48%, respectively. In the 40 ~ 60 cm layer, the reductions were 59.40%, 30.08%, and 63.91%, respectively. At the Dongsheng site, root dry weight in the 0 ~ 20 cm layer was also significantly lower in all transplanting treatments than in CK, decreasing by 24.70%, 10.69%, and 30.91% under 2L1H, 3L1H, and 4L1H, respectively. In the 20 ~ 40 cm layer, only the 2L1H treatment showed a significant reduction compared with CK (16.47% decrease). In the 40 ~ 60 cm layer, root dry weight was significantly reduced under 2L1H and 3L1H treatments, by 45.68% and 30.86%, respectively. These results indicate that transplanting has the greatest impact on root development in the 0 ~ 20 cm soil layer. Differences among transplanting treatments were more pronounced in deeper soil layers (20 ~ 60 cm), and responses varied between varieties and sites.

The root morphological characteristics of maize plants in the 0 ~ 20 cm soil layer were affected by the seedling transplanting age. All morphological indexes showed a downward trend. There were significant differences in root length, surface area, and number of forks, but no significant difference in the average root diameter (**Table 3**). Compared with CK, the root length of 2L1H and 4L1H treatments in Wenduhua test site reduced significantly by 17.45% and 18.09%. The surface area reduced significantly by 19.75% and 20.19%. And the number of forks reduced significantly by 21.01% and 35.69%. In Dongsheng site the root length reduced significantly by 21.60%, 8.70%, and 28.91%. The surface area reduced significantly by 22.90%, 9.23%, and 31.63%. And the number of branches reduced significantly by 21.86%, 16.10%, and 26.28%.

**Table 3:** Root morphological characteristics in 0 ~ 20 cm soil layer of maize under different seedling transplanting ages.

Experimental location	Treatment	Length (m)	Avg Diam (mm)	Surf Area (cm^2^)	Forks
Wenduhua	2L1H	42.68 ± 1.13b	0.99 ± 0.03a	1323.21 ± 28.31b	15492 ± 694b
	3L1H	51.22 ± 2.41a	1.01 ± 0.04a	1624.94 ± 73.78a	16875 ± 798b
	4L1H	42.35 ± 1.49b	0.98 ± 0.03a	1316.04 ± 54.12b	12613 ± 773c
	CK	51.70 ± 2.61a	1.02 ± 0.03a	1648.92 ± 77.80a	19613 ± 790a
Dongsheng	2L1H	50.49 ± 2.04c	1.01 ± 0.01a	1601.33 ± 72.34c	21729 ± 1114bc
	3L1H	58.80 ± 1.16b	1.02 ± 0.06a	1885.12 ± 117.45b	23332 ± 873b
	4L1H	45.78 ± 2.69d	0.99 ± 0.02a	1419.94 ± 58.59d	20500 ± 1014c
	CK	64.40 ± 1.69a	1.03 ± 0.02a	2076.91 ± 63.49a	27809 ± 1497a

The different letters following the numbers in the table indicate significant differences among treatments (p < 0.05). The same as below.

### 3.4. Effects of seedling transplanting age on leaf photosynthetic performance

#### 3.4.1. Leaf photosynthetic morphological characteristics.

**Fig 3:**
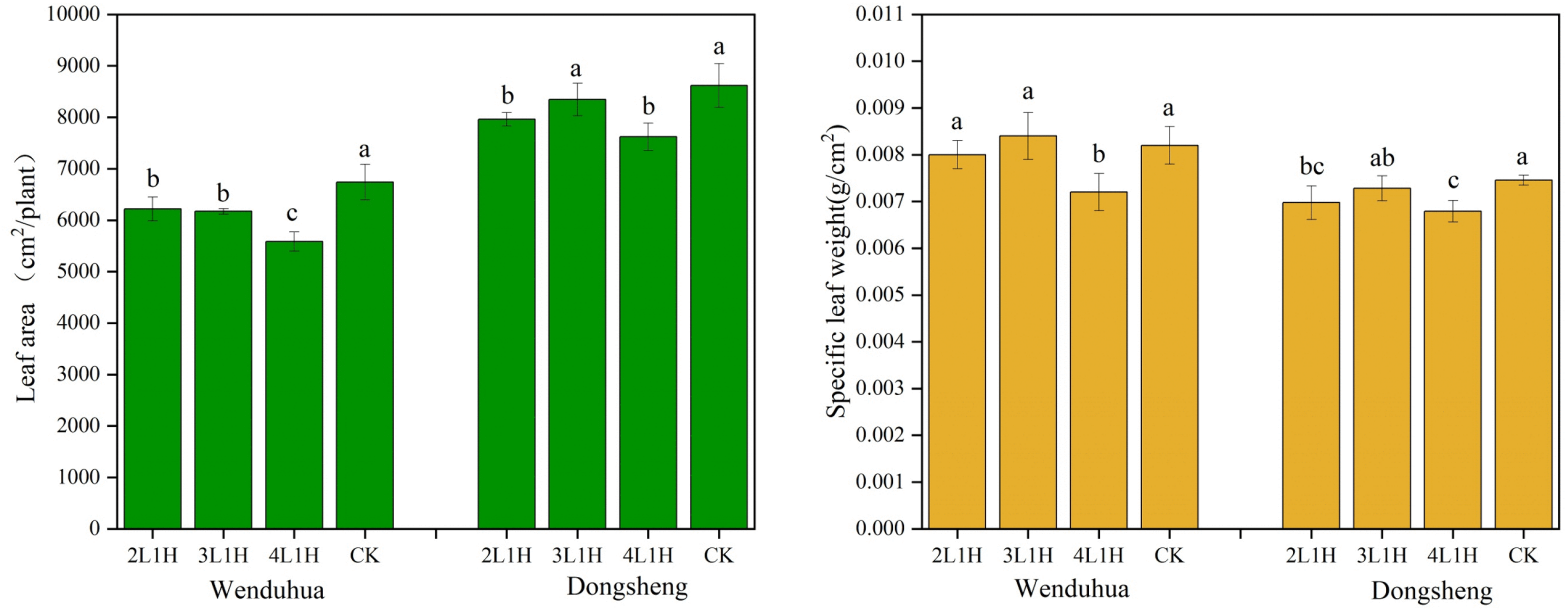
Effects of transplanting age on leaf area and specific leaf weight in plants. Different letters above bars indicate significant differences between treatments (p < 0.05).

**Fig 4:**
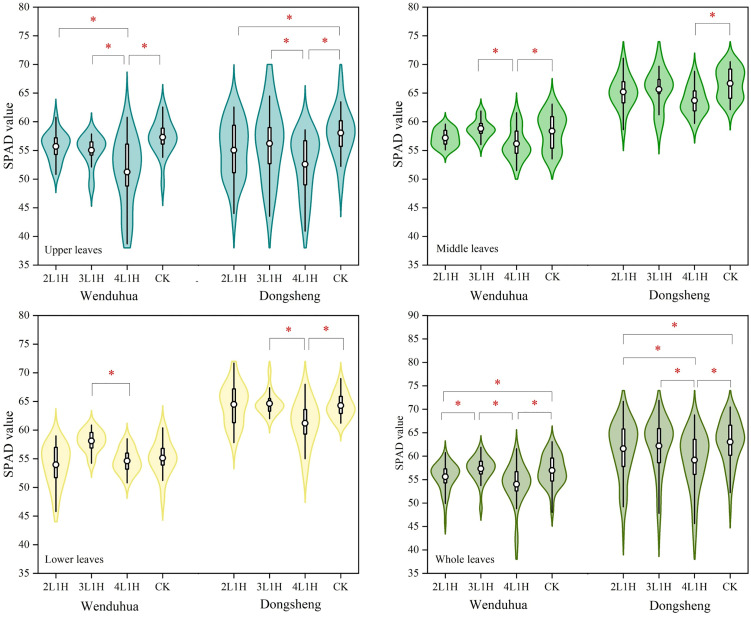
Effects of transplanting age on leaf SPAD values. Upper leaves represent the average of the top three leaves; Middle leaves represent the average of the three leaves around the ear; Lower leaves represent the average of the three leaves near the ground; Whole leaves represent the average of the upper, middle, and lower leaves. * indicate significance at p < 0.05.

Seedling transplanting reduced leaf area, specific leaf weight (SLW), and SPAD values (relative chlorophyll content) in spring maize, with differences observed among treatments (**Figs 3 and 4**). At the Wenduhua site, leaf area per plant (**Fig 3**) was significantly lower in all transplanting treatments than in the control (CK), decreasing by 7.74%, 8.43%, and 17.15% under 2L1H, 3L1H, and 4L1H, respectively. At the Dongsheng site, leaf area in the 3L1H treatment did not differ significantly from CK, whereas the 2L1H and 4L1H treatments showed significant reductions of 7.60% and 11.53%, respectively. For specific leaf weight, at the Wenduhua site, no significant differences were observed between CK and the 2L1H and 3L1H treatments, while the 4L1H treatment showed a significant decrease of 11.76%. At the Dongsheng site, the 3L1H treatment did not differ significantly from CK, whereas the 2L1H and 4L1H treatments were significantly lower than CK, decreasing by 6.44% and 8.92%, respectively.

SPAD values (**Fig 4**) of upper leaves were lower in all treatments compared with CK at both sites. At Wenduhua, the reductions were 2.79%, 3.97%, and 10.60% under 2L1H, 3L1H, and 4L1H, respectively, with a significant difference observed for the 4L1H treatment. At Dongsheng, SPAD values decreased by 5.17%, 3.15%, and 9.39%, respectively, with significant differences in the 2L1H and 4L1H treatments. For middle leaves, only the 4L1H treatment showed significant reductions at both sites, decreasing by 3.79% (Wenduhua) and 4.50% (Dongsheng). For lower leaves, no significant differences were observed among treatments at the Wenduhua site, whereas at Dongsheng, the 4L1H treatment was significantly lower than CK, decreasing by 4.83%. For whole-plant SPAD values, no significant difference was observed between the 3L1H treatment and CK at either site. However, significant reductions were observed in the 2L1H and 4L1H treatments. Compared with CK, SPAD values decreased by 2.23% and 5.43% at Wenduhua, and decreased by 2.27% and 6.11% at Dongsheng, respectively.

#### 3.4.2. Photosynthetic efficiency.

**Fig 5:**
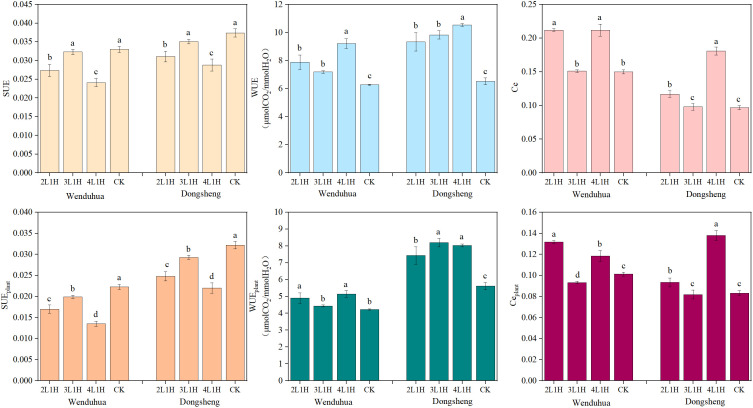
Effects of seedling transplanting age on photosynthetic efficiency during grain filling stage. SUE, instantaneous spectral use efficiency; WUE, instantaneous water use efficiency; Ce, instantaneous carboxylation efficiency; SUE_plant_, plant spectral use efficiency; WUE_plant,_ plant water use efficiency; Ce_plant_, plant carboxylation efficiency. Different letters above bars indicate significant differences between treatments (p < 0.05).

Under transplanting treatment, maize plants exhibited a stress-adaptive response characterized by higher water use efficiency (WUE), achieved by reducing stomatal conductance and thereby limiting leaf water loss. This was accompanied by a decrease in spectral use efficiency (SUE) and an increase in carboxylation efficiency (Ce), indicating partial compensation for reduced CO₂ assimilation (**Fig 5**).

The overall trends of SUE, WUE, and Ce were consistent between the two experimental sites under different treatments. For SUE, the 2L1H and 4L1H treatments were significantly lower than the control (CK), while no significant difference was observed between 3L1H and CK. At the Wenduhua site, SUE decreased by 17.08% and 26.99% under 2L1H and 4L1H, respectively, whereas at the Dongsheng site, the reductions were 16.63% and 22.86%, respectively. In contrast, WUE was significantly higher in all transplanting treatments compared with CK. At the Wenduhua site, WUE increased by 25.53%, 14.49%, and 46.85% under 2L1H, 3L1H, and 4L1H, respectively. At the Dongsheng site, the corresponding increases were 43.31%, 50.82%, and 61.70%, respectively. For Ce, both 2L1H and 4L1H treatments were significantly higher than CK, whereas no significant difference was observed between 3L1H and CK.

From the perspective of whole-plant photosynthetic efficiency, differences in SUEₚₗₐₙₜ between treatments and CK were more pronounced, with reductions of 23.50%, 10.51%, and 39.51% at the Wenduhua site, and 22.97%, 8.84%, and 31.76% at the Dongsheng site for 2L1H, 3L1H, and 4L1H, respectively. In contrast, differences in WUEₚₗₐₙₜ and Ceₚₗₐₙₜ were relatively smaller. At the Wenduhua site, whole-plant WUEₚₗₐₙₜ increased by 15.82%, 4.83%, and 21.71% in the treatment groups compared with CK, while Ce increased by 29.99% and 16.93% under 2L1H and 4L1H, respectively. At the Dongsheng site, WUEₚₗₐₙₜ increased by 32.41%, 46.12%, and 43.04%, respectively, while Ce increased by 12.29% and 66.09% under 2L1H and 4L1H, respectively.

### 3.5. Effect of field transplanting timing on dry matter accumulation and distribution ratio

**Fig 6:**
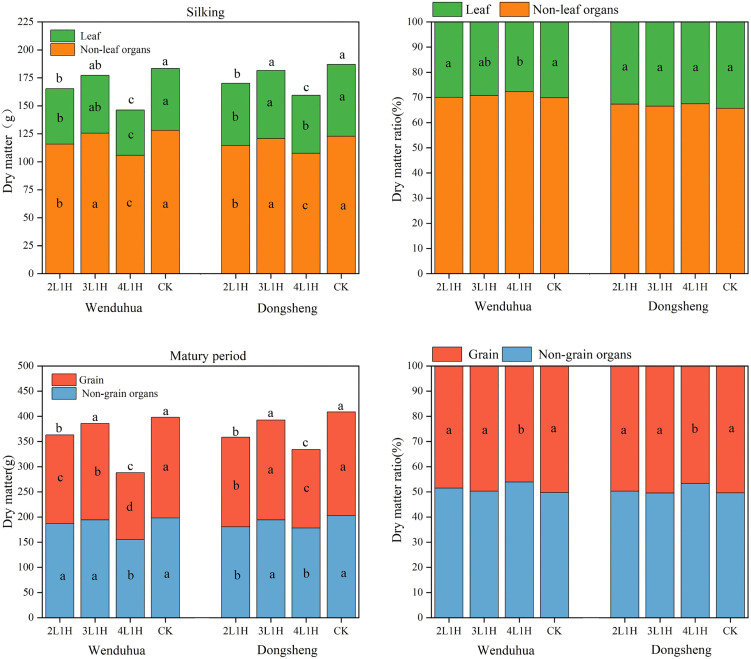
Effects of seedling transplanting age on dry matter accumulation in maize plants. Different letters above bars indicate significant differences between treatments (p < 0.05).

Transplanting reduced maize dry matter accumulation and altered the distribution of dry matter among key organs (**Fig 6**).At the silking stage, total dry matter in the 2L1H and 4L1H treatments was significantly lower than in the control (CK) at both experimental sites, whereas no significant difference was observed between the 3L1H and CK. At the Wenduhua site, compared with CK, dry matter in non-leaf organs decreased by 9.61%, 1.99%, and 17.46% under 2L1H, 3L1H, and 4L1H, respectively. Leaf dry matter decreased by 10.49%, 6.59%, and 26.87%, respectively, while whole-plant dry matter decreased by 9.87%, 3.38%, and 20.30%, respectively. At the Dongsheng site, non-leaf organ dry matter decreased by 6.71%, 1.63%, and 12.39%, leaf dry matter decreased by 13.53%, 5.43%, and 19.38%, and whole-plant dry matter decreased by 9.05%, 2.94%, and 14.79%, respectively. Regarding dry matter distribution, the proportion of leaf to non-leaf organs showed a decreasing trend under transplanting. At the Wenduhua site, the difference between the 4L1H treatment and CK was significant, whereas at the Dongsheng site, no significant differences were observed among treatments.

At physiological maturity, dry matter accumulation in all plant organs showed similar trends. At the Wenduhua site, dry matter in non-grain vegetative organs decreased by 5.53%, 1.96%, and 21.51% under 2L1H, 3L1H, and 4L1H, respectively, with a significant difference observed for the 4L1H treatment. Grain dry matter decreased by 12.06%, 4.21%, and 33.71%, respectively, with all treatments significantly lower than CK. Whole-plant dry matter decreased by 8.81%, 3.09%, and 27.64%, with significant differences observed for the 2L1H and 4L1H treatments. At the Dongsheng site, dry matter accumulation under 2L1H and 4L1H was significantly lower than CK, while no significant difference was observed for 3L1H. Compared with CK, dry matter in non-grain organs decreased by 10.98%, 4.10%, and 12.12%, grain dry matter decreased by 13.65%, 3.70%, and 24.27%, and whole-plant dry matter decreased by 12.32%, 3.90%, and 18.25%, respectively. In terms of dry matter partitioning, the grain-to-non-grain ratio decreased under transplanting at both sites, with a significant reduction observed in the 4L1H treatment, while differences in the 2L1H and 3L1H treatments were not significant. These results indicate that transplanting not only reduces grain dry matter accumulation but also restricts the translocation of assimilates from vegetative organs to grains.

### 3.6. Effect of field transplanting timing on maize ear traits

Transplanting reduced maize ear trait indices (**Table 4**). Significant differences were observed among treatments in ear length, ear diameter, number of kernels per row, 1000-kernel weight, and kernel weight per ear, whereas no significant difference was found in the number of kernel rows per ear. At the Wenduhua site, compared with CK, ear length decreased by 13.71% and 14.71% under the 2L1H and 4L1H treatments, respectively. Ear diameter decreased by 4.55% and 6.82%, number of kernels per row decreased by 10.85% and 11.37%, 1000-kernel weight decreased by 5.96% and 10.67%, and kernel weight per ear decreased by 12.05% and 33.70%, respectively. At the Dongsheng site, ear length decreased by 8.89% and 13.33% under 2L1H and 4L1H, respectively. Number of kernels per row decreased by 5.97% and 13.68%, 1000-kernel weight decreased by 4.46% and 8.42%, and kernel weight per ear decreased by 13.64% and 24.27%, respectively. Overall, reductions in ear traits were more pronounced under the 4L1H treatment than under the other transplanting stages, with kernel weight per ear showing the greatest decline.

**Table 4:** Effects of seedling transplanting age on ear traits of maize.

Experimental location	Treatment	Ear length (cm)	Ear diameter (cm)	Kernel rows	Kernels per row	Kernels weight (g)	Kernels weight per spike (g)
Wenduhua	2L1H	17.24 ± 0.69b	4.15 ± 0.29bc	15.8 ± 0.6a	34.5 ± 2.6b	378.4 ± 3.2b	175.9 ± 6.2c
	3L1H	19.82 ± 0.81a	4.35 ± 0.17ab	15.4 ± 1.9a	38.2 ± 1.8a	397.0 ± 11.4a	191.6 ± 4.5b
	4L1H	17.04 ± 0.67b	4.06 ± 0.26c	15.6 ± 1.3a	34.3 ± 3.0b	368.6 ± 6.1c	132.6 ± 4.9d
	CK	19.98 ± 0.69a	4.41 ± 0.16a	15.6 ± 0.8a	38.7 ± 1.4a	401.5 ± 8.1a	200.0 ± 3.9a
Dongsheng	2L1H	17.03 ± 1.14b	4.14 ± 0.17c	15.4 ± 1.7a	37.8 ± 3.7ab	378.0 ± 7.3b	177.9 ± 4.5b
	3L1H	19.36 ± 1.37a	4.30 ± 0.18b	16.0 ± 1.3a	39.1 ± 3.4a	395.1 ± 8.2a	198.4 ± 6.0a
	4L1H	16.90 ± 1.08b	3.93 ± 0.15d	15.6 ± 0.8a	34.7 ± 3.9b	362.8 ± 6.3c	156.0 ± 7.6c
	CK	19.47 ± 1.74a	4.48 ± 0.12a	16.2 ± 1.1a	40.2 ± 3.9a	404.6 ± 5.1a	206.0 ± 7.9a

### 3.7. Statistical analysis of maize traits under different seedling transplanting age

**Fig 7:**
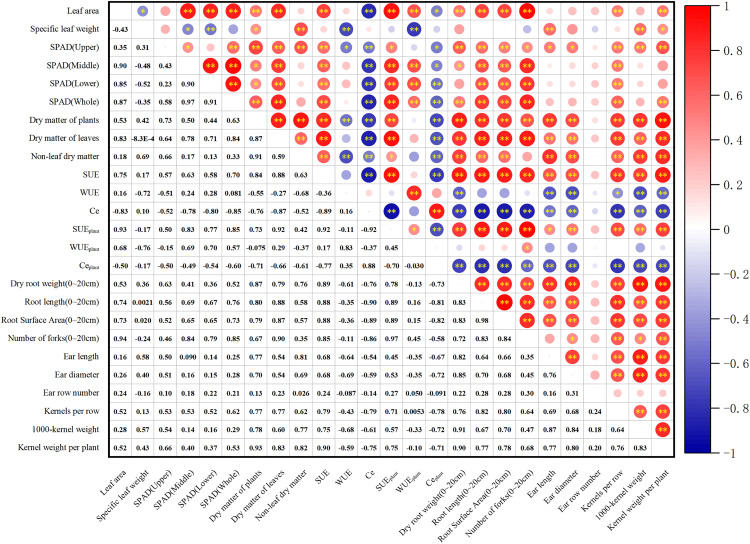
Correlation between root-shoot traits and plant yield. *and** indicate significance at p < 0.05 and p < 0.01.

Correlation analysis was conducted among root–shoot characteristics, physiological traits, and ear traits of spring maize (**Fig 7**). The results showed significant correlations among plant traits at the silking stage (upper leaf SPAD, plant dry weight, leaf dry weight, and non-leaf organ dry weight), photosynthetic efficiency indices (SUE, Ce, SUEₚₗₐₙₜ, Ceₚₗₐₙₜ), root traits (root dry weight, root length, root surface area, and number of forks), and yield-related traits (ear length, ear diameter, number of kernels per row, 1000-kernel weight, and kernel weight per ear). Among these, plant dry weight, SUE, root dry weight, and kernel weight per ear showed the largest correlation coefficients. Leaf area, middle leaf SPAD, and whole-plant SPAD were also significantly correlated with photosynthetic efficiency indices and root traits.

**Fig 8:**
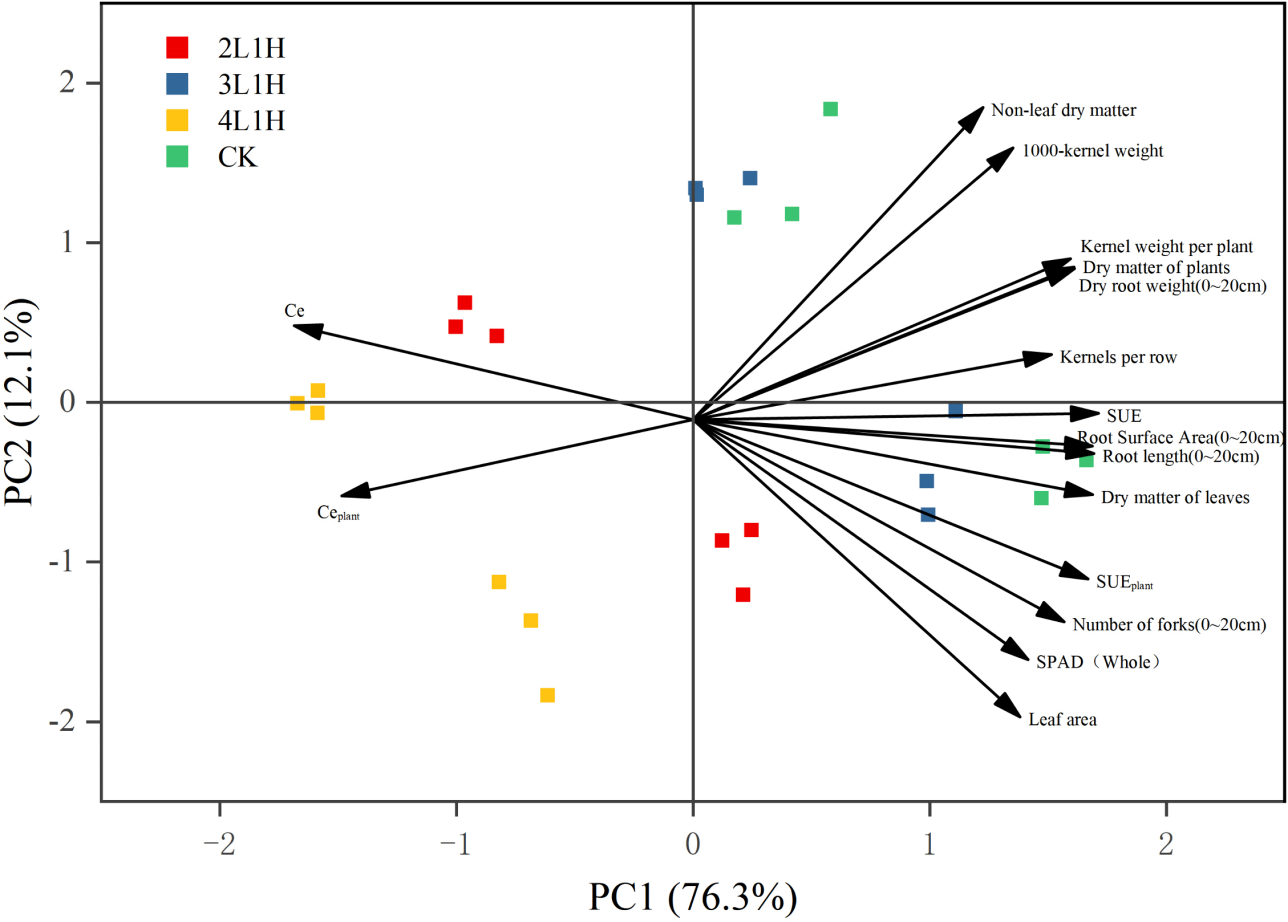
Principal component analysis of maize trait indices under different seedling transplanting ages.

Principal component analysis (PCA) was further performed to evaluate relationships among indices (**Fig 8**). The results showed that the eigenvalues of the first two principal components were greater than 1, with a cumulative contribution rate of 88.4%. The first principal component (PC1) accounted for 76.3% of the total variation, with a weighting coefficient of 0.863. In PC1, most indices showed positive loadings, whereas Ce and Ceₚₗₐₙₜ showed negative loadings. Variables with high loading coefficients included SUE (0.274), root length (0.271), root surface area (0.270), leaf dry weight (0.270), SUEₚₗₐₙₜ (0.267), plant dry weight (0.258), root dry weight (0.258), and kernel weight per ear (0.255), indicating that PC1 mainly reflected integrated root–shoot growth and photosynthetic performance. The second principal component (PC2) explained 12.1% of the total variation, with a weighting coefficient of 0.137. Non-leaf organ dry weight and 100-kernel weight showed positive loadings, whereas leaf area and whole-plant SPAD showed negative loadings. Variables with higher loading coefficients included non-leaf organ dry weight (0.450), leaf area (−0.428), 1000-kernel weight (0.392), and plant SPAD (−0.346), indicating that PC2 mainly represented variation in leaf and ear traits. Based on comprehensive scores, the 2L1H and 4L1H treatments differed significantly from the control (CK), whereas no significant difference was observed between the 3L1H treatment and CK.

## 4. Discussion

Studies have shown that transplanting can extend the crop growth period, and that older seedlings generally result in a longer growth duration [10,20]. Based on this, nursery seedling transplanting is widely used to improve crop productivity. Compared with direct seeding, nursery transplanting eliminates the field germination stage, allowing crops to more effectively utilize light and heat resources from seedling establishment to maturity, thereby enhancing dry matter accumulation and yield, and often advancing maturity [21,22]. However, a longer growth period induced by transplanting older seedlings does not necessarily lead to higher yield; in many cases, yield is reduced. This is mainly because increasing seedling age often prolongs vegetative growth while shortening the effective reproductive growth period, thereby reducing the duration of dry matter accumulation and ultimately decreasing yield. Therefore, optimal yield is achieved only when transplanting is conducted at an appropriate seedling stage [15,19,23,24]. In the present study, compared with direct seeding, transplanting at different seedling ages delayed overall crop development and extended the total growth period. The extension mainly occurred during the seedling-to-jointing stage and the silking-to-maturity stage (reproductive phase), while the duration from jointing to silking (vegetative phase) was shortened. With increasing seedling age, the delay in the seedling-to-jointing stage and the reproductive period became more pronounced, indicating a prolonged “seedling recovery” period after transplanting, which is consistent with previous findings.

The coordinated interaction between root and shoot systems plays a crucial role in crop growth, development, and yield formation [25]. The root system acts as a bridge between the soil and the canopy, supplying water and nutrients to aboveground organs, while the shoot system provides assimilates to support root growth. The continuous exchange of matter and energy between these two components promotes overall plant development [26]. Root morphological characteristics, spatial distribution, and physiological activity determine the plant’s capacity for water and nutrient uptake, thereby influencing stress tolerance and yield potential [27–30]. Well-developed root systems can delay leaf senescence after silking, maintain photosynthetic capacity, enhance post-silking dry matter accumulation, and ultimately improve grain filling and final yield [31].

**Fig 9:**
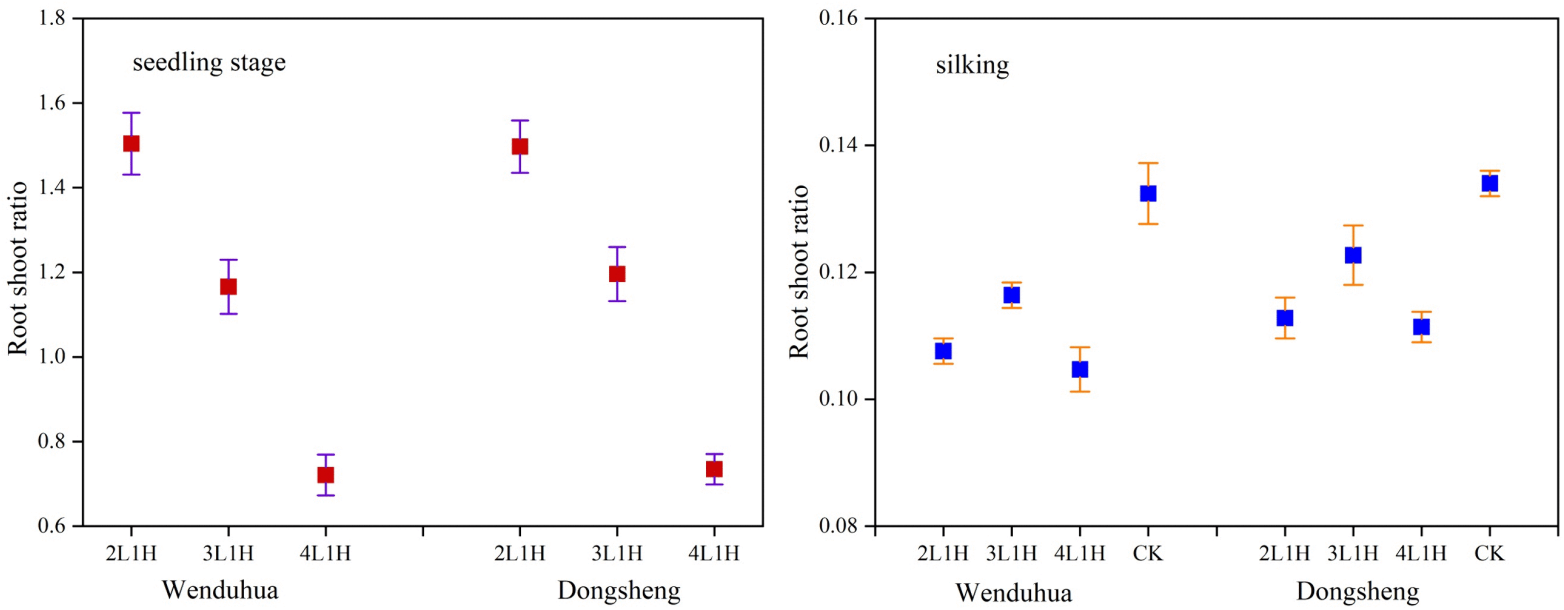
Root-shoot ratio at different growth stages. Root-shoot ratio represents the ratio of root dry weight to aboveground dry weight.

In this study, a shallow drip irrigation system was used, which supplied water and nutrients according to maize growth demand while reducing deep percolation losses and concentrating resources in the shallow soil layer [32]. As a result, maize roots were mainly distributed in the 0 ~ 20 cm soil layer, where more than 90% of root dry weight was concentrated [33]. This root zone was most strongly affected by transplanting and showed a close relationship with canopy traits. Transplanting resulted in reduced root length, root surface area, branching number, and root biomass, as well as an imbalanced root/shoot ratio (**Fig 9**), indicating disrupted coordination between belowground and aboveground growth [15]. Consequently, root “source strength” for resource acquisition was weakened, leading to reduced uptake efficiency. Even under adequate water and fertilizer conditions, plants exhibited a stress-adaptive response: leaf area, specific leaf weight, and SPAD values decreased, total photosynthetic carbon assimilation declined (lower SUE), while carboxylation efficiency (Ce) and water use efficiency (WUE) increased. These changes ultimately reduced dry matter accumulation, impaired ear development, and decreased yield. This mechanism also explains why, despite a longer growth period-particularly the extended reproductive phase observed under transplanting at different seedling ages-yield remained lower than that under direct seeding. In practical production, transplanted seedlings also require a “recovery period,” during which growth is slower than that of direct-seeded plants. Under high planting density, competition pressure further intensifies this imbalance between root and shoot development, exacerbating yield reduction [34].

Previous studies have shown that root traits at the seedling stage play an important role in determining final yield [35], and an appropriate root-to-shoot ratio can help maintain yield under stress conditions [36]. In this study, the root-to-shoot ratio decreased significantly with increasing seedling age (**Fig 9**). Among treatments, 3L1H performed best, showing the smallest differences in photosynthetic performance, dry matter accumulation, and yield traits compared with direct seeding. Therefore, both excessively high and low root-to-shoot ratios are unfavorable for transplanting. A moderate root-to-shoot ratio (approximately 1.1 ~ 1.3) appears to minimize transplanting stress and may serve as an important reference indicator for selecting suitable seedling stages for transplanting.

## 5. Conclusions

Transplanting maize seedlings prolonged the duration of the seedling-to-jointing and silking-to-maturity stages (reproductive phase), while shortening the jointing-to-silking stage (vegetative phase). With increasing seedling age at transplanting, the total growth period showed an overall extension trend. Compared with direct seeding, transplanting mainly affected the formation of canopy photosynthetic characteristics by restricting root system development. This led to reduced photosynthetic efficiency, decreased dry matter production capacity, and altered dry matter partitioning among key organs. Consequently, ear development was impaired and grain yield was reduced. Among the treatments, transplanting at the 3-leaf 1-heart (3L1H) stage minimized yield losses and showed the most favorable overall performance. These results provide a theoretical basis for optimizing on-site transplanting practices of maize under field conditions.

## Supporting information

S1 Data Data. (XLS)
